# Integrating microarray data and single-cell RNA-seq reveals correlation between kit and nmyc in mouse spermatogonia stem cell population

**DOI:** 10.3389/fcell.2025.1634347

**Published:** 2025-08-29

**Authors:** Danial Hashemi Karoii, Hossein Azizi, Thomas Skutella

**Affiliations:** ^1^ Faculty of Biotechnology, Amol University of Special Modern Technologies, Amol, Iran; ^2^ Department of Cell and Molecular Biology, School of Biology, College of Science, University of Tehran, Tehran, Iran; ^3^ Institute for Anatomy and Cell Biology, Medical Faculty, University of Heidelberg, Heidelberg, Germany

**Keywords:** spermatogonial stem cells, KIT gene, spermatogenesis, stem cell differentiation, microarray, pluripotency pathways

## Abstract

Spermatogonial stem cells (SSCs) are essential for the continuous production of sperm and the maintenance of male fertility. Their selection, culture, and molecular characterization provide critical insights into spermatogenesis and potential therapeutic applications for male infertility. This study utilized CD49f-MACS and matrix selection techniques to isolate SSCs from mouse testicular samples. The molecular profile of the selected SSCs was analyzed through immunocytochemistry, gene ontology enrichment, weighted gene co-expression network analysis (WGCNA), and single-cell RNA sequencing (scRNA-seq). Additionally, protein-protein interaction (PPI) networks were constructed to identify key regulatory factors in SSC maintenance and differentiation. The selected SSCs exhibited a distinct molecular signature, with high expression of Dazl, Pou5f1 (Oct4), Gfra1, Nanog, and Kit. The Kit gene (c-kit) emerged as a crucial regulator of SSC differentiation, strongly associated with retinoic acid (RA)-mediated signaling pathways. Co-expression analysis revealed significant interactions between Kit, Nmyc, and other pluripotency-associated genes, highlighting its role in SSC development. Furthermore, single-cell RNA sequencing confirmed the dynamic expression of Kit during SSC differentiation and early meiosis initiation. Our findings underscore the pivotal role of Kit in spermatogenesis, reinforcing its potential as a therapeutic target for treating male infertility. The study also provides a comprehensive molecular framework for understanding SSC biology, with implications for regenerative medicine, fertility preservation, and *in vitro* gametogenesis. Further research integrating gene-editing technologies and *in vivo* models will be essential to explore the full therapeutic potential of SSC-based treatments.

## Introduction

Sperm generation in the seminiferous epithelium is achieved during the process of spermatogenesis, tightly controlled and involving differentiation and intricate morphologic changes (Azizi et al., 2022). The three primary steps in adult testis are the proliferation of spermatogonial cells (Spg), meiosis of spermatocytes, and spermiogenesis of haploid spermatids (Hashemi Karoii and Azizi, 2023). Spermatogenesis in adult testes begins with diploid SSCs. Seminiferous tubule basal membranes include SSCs, which are also known as type As Spg. Both the As Spg and the Ap Spg self-renew (Davoodi Nik et al., 2024). The Ap Spg is differentiated into chains of 4, 8, or 16 type Aal Spg after each division, and then moves along the basal membrane. Morphological criteria have traditionally referred to the SSCs, Ap, and Aal Spg as undifferentiated Spg. After a final mitosis, the Aal Spg is differentiated into a more committed A1 Spg, which is divided and differentiated into intermediate A4, A2, A3, and B Spg. The expression of c-kit differs between the “differentiating” (A1, A2, A3, A4, intermediate, and B) Spg and the “undifferentiated” (As, Ap, and Aal) Spg. Gain of Kit is timed to correspond with the change from undifferentiated Spg to differentiating Spg (Zhang et al., 2011). Identifying Spg has been commonly performed by looking for Kit in Spg. Essential functions for the survival of cells expressing Kit are related to Kit, which is expressed until meiosis (Zhang et al., 2013).

A protein called Kitl is produced by Sertoli cells, which tightly surround the germ cells in the epithelium (Yi et al., 2025). The Kit/Kitl pathway is believed to be essential for the development, migration, survival, and maturation of germ cells in both the embryonic and postnatal gonads, since Kit can only be activated upon interaction with Kitl (Yang et al., 2024). The round-to-elongating spermatid transition is disrupted in mice with a mutation that makes Kit kinase constitutively active. Four recognized pathways are active when Kit or Kitl are activated in the Spg (Shams et al., 2025). The PI3K pathway is responsible for cell survival, adhesion, and proliferation by regulating AKT and BAD, activating c-JUN and c-FOS, and AKT and p70S6K, respectively. Only at the postnatal stage of spermatogenesis does the PI3K/AKT pathway play a crucial role. Mice without Kit are sterile due to decreased proliferation and higher apoptosis in the Spg, and they cannot recruit PI3K (Chen et al., 2022). It has been suggested that cyclin may be one of the targets of the Kit/Kitl pathway, since the PI3K pathway facilitates it and leads to overexpression and nuclear accumulation of cyclin D3 and Spg proliferation. Furthermore, in mouse PGCs, cell migration, and AKT phosphorylation are influenced by the SRC pathway, which is comprised of SRC family members interacting with Kit’s intracellular juxtamembrane domain (Beumer et al., 2000). Thirdly, the PLCG pathway, which Tr-Kit initiated, allows the fertilized eggs to resume meiosis. The MAPK cascade is finally triggered by RAS upon Kit and GRB2 binding. In both PGCs and Spgs, MAPK has a direct mediating role in gene transcription (Blücher et al., 2023; Amirian et al., 2022).

## Materials and methods

### Digestion and culture of testicular cells

The testis of 7-week-old C57BL/6 mice were used to create SSCs. We killed the newborn mouse and removed its testes through a tiny incision in its belly button. After being used in the experiment, adult mice were killed by carbon dioxide. Using a two-step mechanical and enzymatic digestion solution, which included 0.05 mg/mL DNase, 0.5 mg/mL collagenase, and 0.5 mg/mL dispase in an HBSS buffer (PAA, United States), the testis was decapsulated and the tissue was mechanically dissected and dissociated after 8 min at 37 °C with shaking and pipetting. To create a single-cell suspension, 10% ES cell-qualified FBS was pipetted up and down to block the digestion enzymes. After the specimens were spun in a centrifuge for 10 min at 1,500 rpm, they were rinsed with DMEM/F12, passed through a 70 μm cell strainer, and then returned to the rotating apparatus. After removing the supernatant, the testicular cell solution was spread onto culture plates covered with 0.2% gelatin. Cells cultured in the mouse GSC (mGSC) medium consisted of StemPro-34 medium, 1% L-glutamine (PAA, United States), and 1% N2-supplement (Invitrogen, United States). All animal procedures were conducted in compliance with the guidelines of the Institutional Animal Care and Use Committee (IACUC) of [Your Institution Name] and adhered to international ethical standards for animal research. Mice were humanely euthanized using carbon dioxide (CO_2_) inhalation, following a gradual displacement method with a CO_2_ flow rate of 20% of the chamber volume per minute to minimize distress. Upon the cessation of respiration, death was confirmed by a secondary method (cervical dislocation).

In experiments requiring chemical euthanasia, pentobarbital was administered intraperitoneally at a dosage of 150 mg/kg. The animals were monitored for complete loss of consciousness before confirmation of death. All procedures were performed by trained personnel, and appropriate disposal of animal remains followed institutional biosafety protocols.

## Anesthesia and animal handling

No anesthesia was used in the procedures involving newborn mice, as euthanasia was performed before any experimental procedures. For adult mice, chemical euthanasia was conducted using pentobarbital (150 mg/kg, intraperitoneally) for specific experimental conditions requiring deep sedation. In cases where CO_2_ euthanasia was applied, it was performed using a gradual displacement method, as specified above, ensuring minimal distress. No other forms of anesthesia were utilized in this study.

### Selection and cultivation of mGSCs

The mouse testicular tissues were carefully manipulated to separate the tubules after the tunica albuginea was removed. The following enzymes were used to degrade tubules in every sample: 5 μg/mL of DNase, 0.25 mg/mL of dispase II (Roche, Basel, Switzerland), and 750 U/mL of collagenase type IV (Sigma, Darmstadt, Germany). The experiment was conducted for 25 min at 37 °C with moderate agitation in HBSS buffer containing Ca++ and Mg++ (PAA, Farnborough, United Kingdom). The goal was to make a suspension of a single cell. Next, 10% fetal bovine serum (FBS) from Thermo Fisher Scientific in Bremen, Germany, which is qualified by ES cells, was added to the mixture to slow down digestion. The cell cultures that performed the best were subcultured every two to 3 weeks at a 1:2 interval. It was critical to keep the well cell counts at an ideal level at all times to avoid cell dilution.

### Immunocytochemical (ICC) staining

After being treated with 4% paraformaldehyde, the cells were grown on 24-well plates. After a thorough washing with PBS, the samples were treated with a 0.1% Triton/PBS permeabilizer and a 1% BSA/PBS blocking solution. The cells were treated with primary antibodies overnight after the blocking solution was removed. Following the 29th rinse, the procedure was incubated with secondary antibodies specific to the species that had been conjugated with various fluorochromes. After being cross-stained with 0.2 μg/mL of DAPI (4′, 6-diamidino 2-phenylindole) for 3 min at room temperature, the labeled cells were fixed using Mowiol 4–88 reagent. For all markers, a negative control was performed by removing each primary antibody from the sample. A confocal Zeiss LSM 700 microscope was used to observe the labeled cells, and a Zeiss LSM-TPMT camera was used to collect photographs (Shams et al., 2025; Hashemi Karoii et al., 2025a; Hashemi Karoii et al., 2023; Hashemi Karoii et al., 2022; Hashemi Karoii et al., 2024a; Hashemi Karoii et al., 2024b; Hashemi Karoii et al., 2024c; Hashemi Karoii et al., 2025b; Karoii et al., 2022; Niazi Tabar et al., 2022).

### Electron microscopy

The process of electron microscopy began with the pelleting and fixation of both undifferentiated and differentiating spermatogonia in a paraformaldehyde/glutaraldehyde solution that had been buffered with PIPES. After a 5-min initial fixing, the fixative was reapplied to guarantee the best possible preservation. After leaving the pellets to post-fix in a potassium hexacyanoferrate and osmium tetroxide (OsO4) solution for 50 min, they were rinsed in a pH 6.0 sodium maleate buffer and a uranyl acetate buffer. Uranyl acetate was used to block-stain the samples. Beginning with short 5-min treatments and progressing via four lengthy 20-min stages, the tissue was dehydrated using a graded ethanol series. Polymerization was carried out at 58 °C for 24 h after the samples were immersed in Epon resin. By using a Zeiss EM10 electron microscope (Carl Zeiss, Oberkochen, Germany), ultrathin slices of about 50 nm in thickness were created and examined.

### Labelling probes and preparing RNA

The quality and amount of the RNA preparations were evaluated using gel electrophoresis and UV spectrophotometry. Fifty milligrams of total spermatozoal RNA, oligo (dT), M-MLV reverse transcriptase, and two hundred microCi of alpha-33PdATP were added to a reverse transcription solution containing 33P-labeled dATP to produce the cDNA probes (NEN Life Science, Boston, Mass).

### Microarray analysis

The RNA from sperm was isolated using Qiagen’s RNeasy Mini Kit and then amplified using Ambion’s MessageAmp aRNA Kit. After 200 cells per probe were harvested, RNA direct lysis solution was added to each sample and incubated at −75 °C for 12 h. The samples were sent to the Microarray Facility at the University of Tübingen Hospital for testing. We used the Affymetrix mouse U133 + 2.0 Genome Oligonucleotide Array to analyze gene expression. The Berlin, Germany-based MicroDiscovery GmbH was responsible for the normalization and biostatistical analysis (Table 1). In addition, we use public GEO database GSE211115, GSE147982 (Abe et al., 2020), GSE118846 (Kitadate et al., 2019), GSE102214 (Jauregui et al., 2018), GSE77309 (Gopinathan et al., 2017), and GSE98991 (Chan et al., 2017).

**TABLE 1:** GEO Microarray analysis detail.

Dataset/Source	Use in study	Sample size/Content	Platform/Notes
GSE211115	scRNA-seq reference for testis development atlas	∼60,000 cells after QC	Mouse Testis Development Atlas; Seurat used for clustering and annotation
GSE147982	Validation of germ cell markers and gene expression	Public scRNA-seq dataset of mouse spermatogenesis	Used for comparative analysis and validation
GSE118846	Marker validation and co-expression network references	Single-cell transcriptome data from mouse testis	Used for additional gene enrichment comparisons
GSE102214	Stem cell-related expression profile reference	Mouse spermatogonia and early germ cells	Used for comparative expression analysis
GSE77309	Additional SSC gene expression validation	Enriched SSC populations from mice	Used for validating key SSC markers
GSE98991	Network validation and pathway analysis	Transcriptomic profiling of mouse testis cell types	Used for enrichment and co-expression analysis

For the public single-cell RNA-seq dataset GSE211115, standard preprocessing was performed using the Seurat v4.3.0 pipeline. Quality control filtering excluded cells with <200 or >6,000 detected genes and those with >10% mitochondrial gene content to remove low-quality or stressed cells. Batch effect correction across samples was conducted using CCAN implemented in Seurat’s FindIntegrationAnchors () and IntegrateData () functions. Cells were clustered using the Louvain algorithm, with resolution parameters ranging from 0.2 to 1.2 tested. A resolution of 0.6 was chosen based on inspection of the clustering tree, silhouette scores, and consistency with known germ cell and somatic populations. Marker gene expression (e.g., Stra8, Gfra1, Kit) was used to annotate clusters. These steps ensured biologically meaningful and reproducible cell classification.

### Differential expression analysis and data processing

Using a microarray, 50,000 transcripts were examined in non-obstructive azoospermia. Next, these genes are compared to those in typical cells. In the default parameters, the fold change (log2FC) is set at 1.5 and the adj. Value is 0.05. Volcano maps and heat maps are great ways to see differential expression data. R was used to generate the heat map and volcano map.

### Gene expression analyses on the Fluidigm Biomark system

Using the Fluidigm Biomark system, we looked at the Kit and Nmyc gene expression levels in SSCs, ESCs, ES-like cells, and Epiblast cells. A housekeeping gene called glyceraldehyde-3-phosphate dehydrogenase was used for standardization. We used a micromanipulator technique to collect SSCs, ESCs, ES-like, and Epiblast cells. Then, we lysed them with a lysis buffer solution that included 9 µL of RT-PreAmp Master Mix, 5.0 µL of Cells Direct 2× Reaction Mix from Invitrogen, 2.5 µL of 0.2× assay pool, 1.3 µL of TE buffer, and 0.2 µL of RT/Taq Superscript III from Invitrogen, United States of America. Then, using a TaqMan real-time PCR on a BioMark Real-Time Quantitative PCR (qPCR) instrument, the quantity of the amplified product of RNA-targeted copies was evaluated. Two technical replicates were performed on the samples. In order to determine the Ct values, Excel and GenEx were used.

### PPI network construction and analysis

We used the Search Tool for the STRING version 11.5 (Retrieval of Interacting Genes/Proteins database, https://string-db.org/) to create the PPI network. The goal of the online database known as STRING is to compile all anticipated and known protein interactions, including physical and functional correlations. Specifically, the PPI network was built using the STRING program in Cytoscape Software (v 3.10.2). A network was generated using the top 100 proteins in the “Stem cell” in *Mus musculus* using the STRING: PubMed query as the data source, with a minimum confidence score cut-off of 0.70. Then, we built a separate network for the nodes that were initial neighbors with c-KIT, Mycn, Nanog, Dazl, and Pou5f1.

### Gene enrichment analysis

We have used the STRING Enrichment analysis in the Cytoscape Software (https://cytoscape.org/) to look at the roles of certain genes in the sub-network and where they are located in various tissues. We hand-picked fourteen genes associated with c-KIT, Mycn, Nanog, Dazl, and Pou5f1. We did not take a particular FDR value into account when we investigated various functional enrichments in relation to our laboratory data.

#### Weighted gene co-expression network analysis (WGCNA)

Gene co-expression modules connected to hub gene co-expression were found using the intricate WGCNA method. The WGCNA R program has been used to create gene co-expression networks using SSCs DEGs. To summarize, a co-expression module was established via cluster analysis and a weighted correlation adjacency matrix for genes exhibiting similar expression patterns. A appropriate soft threshold β was calculated in order to generate a weighted adjacency matrix and meet the requirements of a scale-free network. A topological overlap matrix (TOM) was generated from the weighted adjacency matrix, and the corresponding dissimilarity was determined by subtracting 1-TOM. Modules were identified using the dynamic tree cutting approach. Merged modules were those with dissimilarities of less than 0.3. When looking for a connection between module eigengene values and immune cell abundance, we used Pearson’s correlation analysis. We chose to focus on modules connected to the signaling pathway that showed a high degree of correlation with the bulk of gene co-expression.

#### Gene co-expression analysis

The following genes were found by a weighted gene co-expression analysis utilizing Coxpressdb and Genemania: c-KIT, Mycn, Nanog, Dazl, and Pou5f1. In order to find gene-to-gene connections, several methods were used to detect gene co-expression networks. For each gene pair, Pearson’s correlation analysis was used to create a similarity matrix. Afterwards, a scale-free co-expression network was constructed utilizing a matrix and an ideal soft threshold power β. Following that, a similar matrix was transformed into a TOM. By summing up all of a gene’s adjacency to all other genes generated by the network, this Transcriptome Overlap Measure (TOM) measures the degree of a gene’s network link. With a minimum gene group size of 50 for the gene dendrogram, we simultaneously assessed the average linkage of hierarchical clustering using the TOM-based dissimilarity measure. A more thorough examination of the module was carried out by computing the dissimilarity of its eigengenes.

#### Data collection of scRNA-seq:

*Mus musculus* is one of nine items included in the first edition of the Male Health Atlas (MHA). The other five organs or tissues are the testis, epididymis, vas deferens, corpus cavernosum, and prostate. There are eight different kinds of cells, and there are 245,263 distinct scRNA profiling (GSE211115 (Zhao et al., 2023)). Our dataset, which includes germ cell lines ranging from spermatogonial stem cells to spermatids, is a subset of the “Mouse Testis Development Atlas.” We provide both basic and advanced clustering classifications based on this dataset. You may access the data in the GEO database by requesting it from GSE211115. When processing the data, we used the limma R package for bulk RNA-seq and the Seurat R package for scRNA-seq. We used a variety of approaches for cell filtering, normalization, dimensionality reduction, and clustering. One of these methods was canonical-correlation analysis (CCA), which we used to eliminate batch effects. After going through quality control, 60,000 cells were kept out of 62,233. UMAP and resolution were chosen for further study. Previous studies’ marker genes were used for cell type annotation. This scATAC-seq data set was generated using the Signac program and the 10X Genomics Cell Ranger ATAC workflow. A total of 850 cells met the stringent quality criteria. Using the FindTransferAnchors tool, scRNA-seq cell types served as the basis for annotating scATAC-seq cell types. Filtering was done using measured genes and unique molecular identifiers (UMIs), and then the spatial transcriptome data was displayed using the Seurat software.

### Statistical analysis

The experiments were repeated no less than three times. We used one-way analysis of variance (ANOVA) with Tukey’s *post hoc* testing to cover each group, and we averaged the gene expression levels across all of them. A non-parametric Mann-Whitney U test was used to compare gene expression. A statistically reliable variance between groups was defined as a value of P < 0.05. Using appropriate databases or web-based data analysis tools, PPI networks were examined.

## Results

### Mouse spermatogonia selection

**FIGURE 1:**
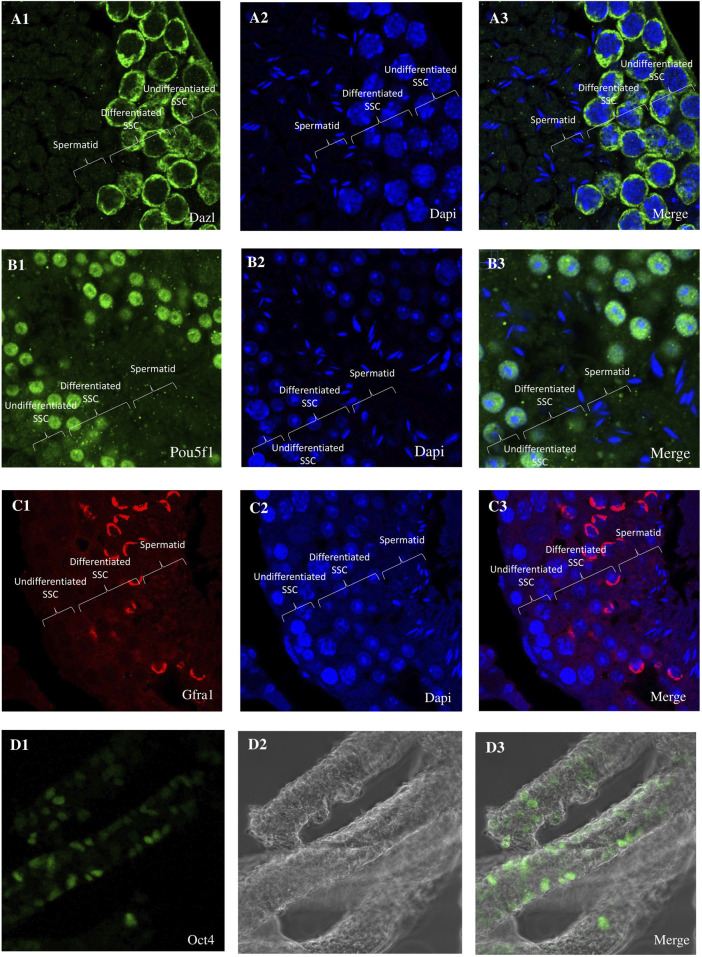
Immunohistochemistry (IHC) and Immunohistochemistry analysis of testis cross-sections. **(A1)** Immunocytochemistry (ICC) reveals the expression of Dazl **(A2)** with DAPI nuclear counterstain in undifferentiated spermatogonia. **(A3)** Merge Dazl and Dapi. **(B1)** Immunocytochemistry (ICC) reveals the expression of Pou5f1 **(B2)** with DAPI nuclear counterstain in undifferentiated spermatogonia. **(B3)** Merge Pou5f1 and Dapi. **(C1)** Immunocytochemistry (ICC) reveals the expression of Gfra1 **(C2)** with DAPI nuclear counterstain in undifferentiated spermatogonia. **(C3)** Merge Gfra1 and Dapi. **(D1)** Immunocytochemistry (ICC) reveals the expression of Gfra1 **(D2)** with DAPI nuclear counterstain in undifferentiated spermatogonia. **(D3)** Merge Gfra1 and Dapi. **(D1)** Immunohistochemistry reveals the expression of Pou5f1 **(D2)** with DAPI nuclear counterstain in undifferentiated spermatogonia. **(D3)** Merge Pou5f1 and Dapi.

**FIGURE 2:**
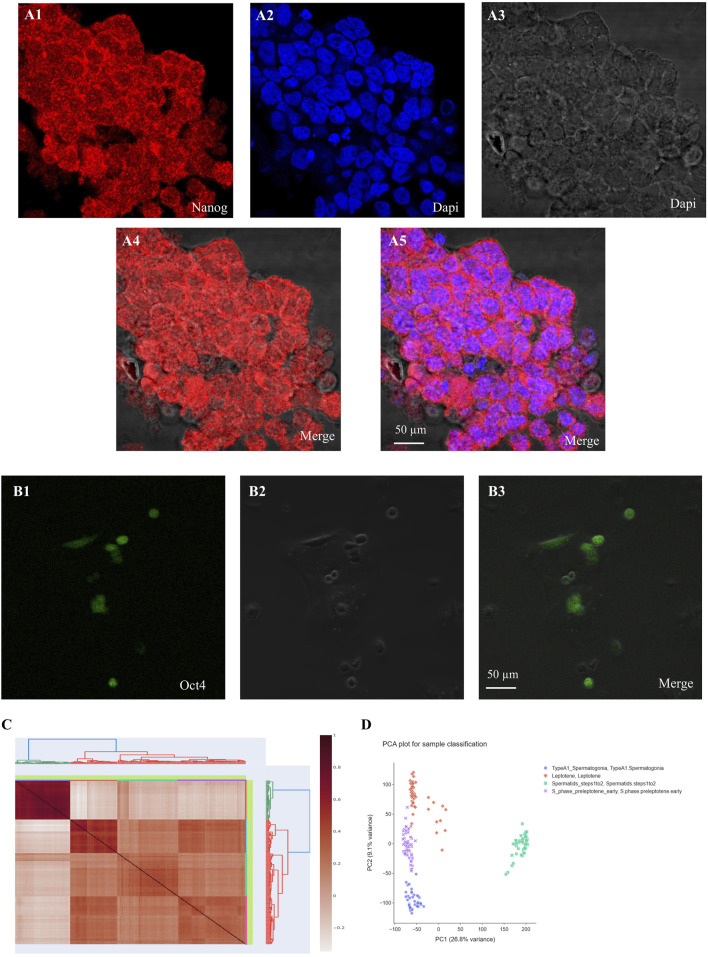
Immunocytochemistry (ICC) reveals the expression of Nanog and Oct4 (green or red) with DAPI nuclear counterstain in undifferentiated spermatogonia. **(A)** Nanog and **(B)** Oct4 show robust expression in undifferentiated spermatogonia, significantly diminishing upon differentiation. The merged images display the colocalization of these gene markers with nuclear staining. **(C)** Correlation plot of SSCs and Spermatid, **(D)** PCA plot for differentially expressed genes based on microarray analysis.

From orchiectomies performed to collect patient data pertinent to SSC cultures, spermatogonia were isolated and concentrated using CD49f-MACS and matrix selection, specifically collagen nonbinding/laminin binding. Positive results for Dazl, Pou5f1, Gfra1, and Oct4 immunocytochemistry in the first tissue suggest that spermatogonia were the most abundant cell type in the chosen cell populations (Figure 1). No matter the age of the patients or the duration of culture, the shape of pure spermatogonia obtained from mouse remained constant. The main reason for this was the spherical shape, together with its size of around 6–12 μm and the high ratio of nucleus to cytoplasm. A bright cytoplasmic ring between the round nucleus and the outside cell membrane is a telltale sign of this ratio. Intercellular bridges allowed all cell cultures to display spermatogonia in pairs, chains, and small clusters. The cultures included many kinds of cells, including larger ones with a diameter of 12–14 μm. There was a smaller nucleus-to-cytoplasm ratio and the cells were oval in shape. There was a significant decrease in mFibs in the unselected cell population. The mFibs were effectively isolated from the nonselected cell fractions after they showed significant proliferation in primary cell cultures. Figure 2 shows spermatogonial cultures that do not include mFibs.

### Stem cell PPI network and sub-network enrichment analyses

**FIGURE 3:**
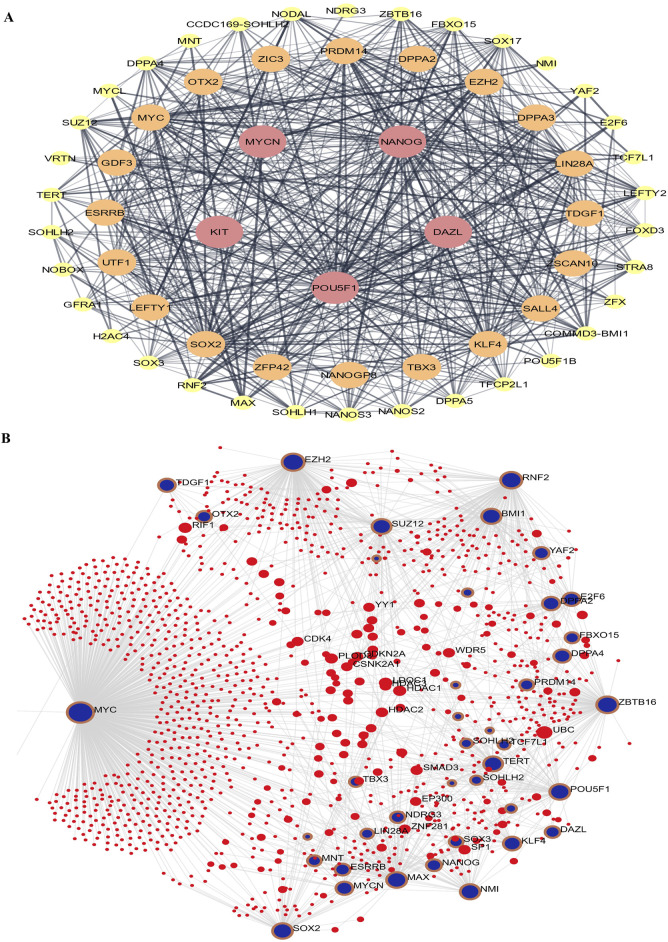
The protein-protein interaction (PPI) Network analysis of stem cell proteins. **(A)** Cytoscape implemented the network based on the STRING PubMed query. **(B)** The maximum number of proteins was set as 100 with a confidence score cut-off >0.40. The red node represents NANOG.

Using STRING and Cytoscape, the “Stem cell” PPI network was mostly composed of 50 nodes and 236 edges. The proteins are represented by the nodes, while the relationships between proteins are shown by the edges. Kit is one of the nodes, indicated in red (Figure 3), according to the network, Dazl, Pou5f1 (Oct4), and Nanog. Using NANOG, we created a sub-network by analyzing the neighboring nodes (Figure 3). There are 126 nodes in this subnetwork. This suggests that there is a substantial correlation between Kit and Nmyc and other stem cell-related genes. To evaluate the molecular roles and cellular locations associated with Kit and Nmyc, we performed STRING Enrichment analysis on ten randomly selected genes. Embryonic development, stem cell differentiation, and stem cell population maintenance are the specific biological processes that we have selected for our experimental investigations. Also, by using TISSUES analysis, one may ascertain the gene location in embryonic stem cells, blastocysts, ectoderm, spermatogonium, adult stem cells, embryoid bodies, and trophectoderm. Another thing we found out using KEGG Pathways is that our genes are part of the signaling networks that control stem cell pluripotency. Furthermore, we show the cellular localization of our genes using COMPARTMENTS analysis (Figure 3).

### Functional enrichment analysis of DEGs

**FIGURE 4:**
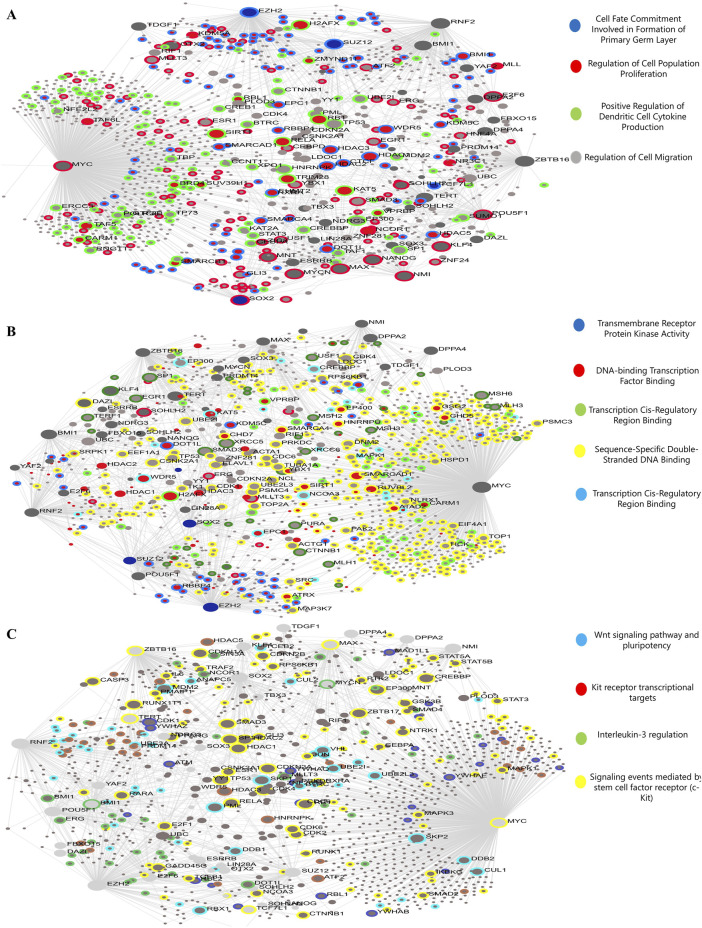
The genes inside the module will be analyzed using gene ontology (GO) enrichment. The quantities of genes are shown by the size of the dots, and the colors indicate the corrected p-values (BH). These images represent **(A)** biological mechanisms, **(B)** molecular activities, and **(C)** the signal transduction network.

Gene ontology enrichment analysis revealed five key terms within the biological process category. These terms included Cell Fate Commitment Involved in primary germ layer formation, regulation of cell population proliferation, positive regulation of dendritic cell cytokine production, and regulation of cell migration. Upregulated and downregulated DEGs were associated with these processes. Upregulated and downregulated differentially expressed genes (DEGs) in the MF category were linked to activities involving transmembrane receptor protein kinase, DNA-binding transcription factors, transcription cis-regulatory regions, and sequence-specific double-stranded DNA binding. Figure 3 shows that signaling events mediated by c-Kit, interleukin-3 regulation, pluripotency, the Wnt signaling pathway, and Kit receptor transcriptional targets were the most abundantly up-and downregulated differentially expressed genes (DEGs) in the signaling pathway (Figure 4).

### Construction of weighted gene Co-Expression modules

**FIGURE 5:**
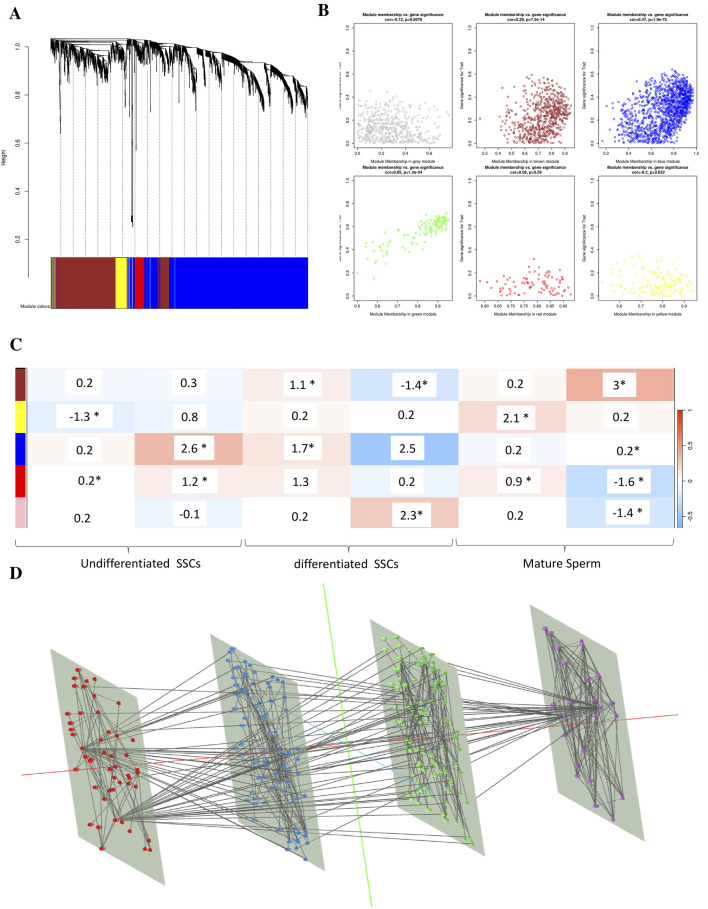
Clinical data in SSCs is used to identify modules. **(A)** A cluster dendrogram of co-expression network modules is generated, with each module being assigned a different hue, using hierarchical gene clustering based on 1-TOM. **(B)** The module and trait relationships are displayed: **(C)** A color-coded module is represented by each row, while clinical characteristic SSCs are denoted by each column. In every cell, you can see the correlation value and the p-value that go along with it (* means it is a significant p-value <0.05). **(D)** The signaling pathway (purple), WGCNA (red), BP (blue), and MF (green) in combination.

In order to find functional clusters in SSCs, we built gene co-expression networks using the WGCNA technique. Five separate modules in the SSC, each given its own color, were recently discovered. It must be noted that the color gray was used to indicate a single module that was not included in any cluster. Then, to assess the relationship between modules and characteristics, we made a heatmap. The connections between modules and characteristics are shown in Figure 5. Figure 5 shows that out of the two modules in the SSC, the brown one has the strongest relationship with normal tissues (r = 0.68, p = 8 × 10^−51^ for the brown module and r = 0.9, p = 1 × 10^−10^ for the pink module) (Figure 5).

### Analysis of single-cell RNA sequencing kit signaling pathways

**FIGURE 6:**
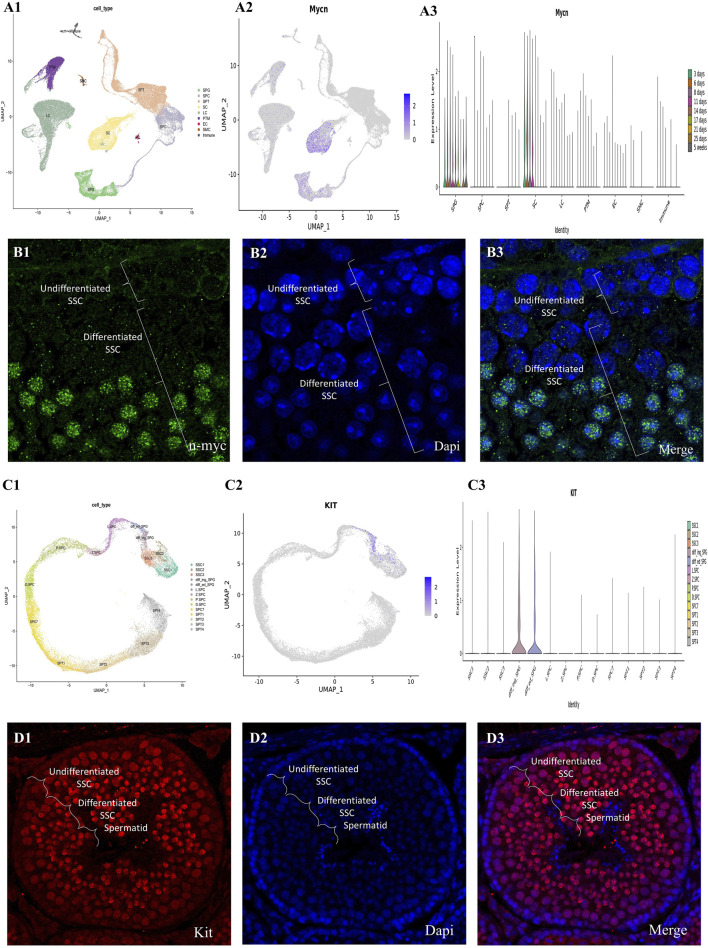
The mouse testis single-cell transcriptome and germ cell lineage transcriptome have been thoroughly investigated. (A1) The UMAP map shows testicular cells that are created from integrated single-cell RNA sequencing data, and it covers periods of testicular development from before birth to after birth. Each of the seventeen color-coded clusters represents a different somatic or germ cell lineage, and there are a total of sixty-3.632 testicular cells in these clusters. (A2) The Nmyc expression patterns of key gene markers for somatic and germ cell lineages are shown in color-coded UMAP plots. Testicular cells are also grouped according to their age in the UMAP plots (A3). Here, W stands for embryonic weeks, D for neonatal days, and Y for years. Fifteen separate clusters were found by the concentrated re-clustering of 8,140 germ cells; these clusters stood for various phases of spermatogenesis, such as Undiff SPG, diff SPG, SCytes, SPtids, and spermatocytes. Germ cell clusters in UMAP are also color-coded according to age, and the expression patterns of important markers for spermatogonia, spermatocytes, and spermatids are reflected in the plots. (C) Dedifferentiated spermatogonia have strong Nmyc expression, which decreases dramatically with differentiation. These gene markers colocalize with nuclear staining, as shown in the combined pictures. (D1) Transforming prenatal to postnatal testicular development into testicular cells, the UMAP plot shows data from integrated single-cell RNA sequencing. Each of the seventeen color-coded clusters represents a different somatic or germ cell lineage, and there are a total of sixty-3.632 testicular cells in these clusters. (D2) The Nmyc expression patterns of key gene markers for somatic and germ cell lineages are shown in color-coded UMAP plots. (D3) In addition, the UMAP plots group testicular cells according to their age, which is represented by W for embryonic weeks, D for neonatal days, and Y for years. Fifteen separate clusters were found by the concentrated re-clustering of 8,140 germ cells; these clusters stood for various phases of spermatogenesis, such as Undiff SPG, diff SPG, SCytes, SPtids, and spermatocytes. Germ cell clusters in UMAP are also color-coded according to age, and the expression patterns of important markers for spermatogonia, spermatocytes, and spermatids are reflected in the plots. (C) Expression of the kit is strong in differentiated spermatogonia but drops down sharply after differentiation. These gene markers colocalize with nuclear staining, as shown in the combined pictures.

We performed pairwise DEG analysis across age groups, merged the five clusters that indicated SSC/progenitors into one, and then used GSEA to do pathway enrichment analysis in order to determine functional changes that occurred during SSC development. With a strict criterion of less than 0.05 for the false discovery rate, the enriched pathways were identified. Figure 6 shows that, in contrast to the newborn phases, the GSEA signature gene collection connected to Kit and n-myc targets is much more abundant during the prenatal stages. Consistent with previous results from mouse PGCs, the abundance of Kit-associated genes is in agreement with the metabolic needs essential for the specification of PGCs from pluripotent stem cells in mice. When we compared the D2_D7 (neonatal stage) to 1 year of age, we found that Kit-associated gene enrichment was preserved (Supplementary Material 1).

### Performance of the fluidigm real-time RT-PCR

**FIGURE 7:**
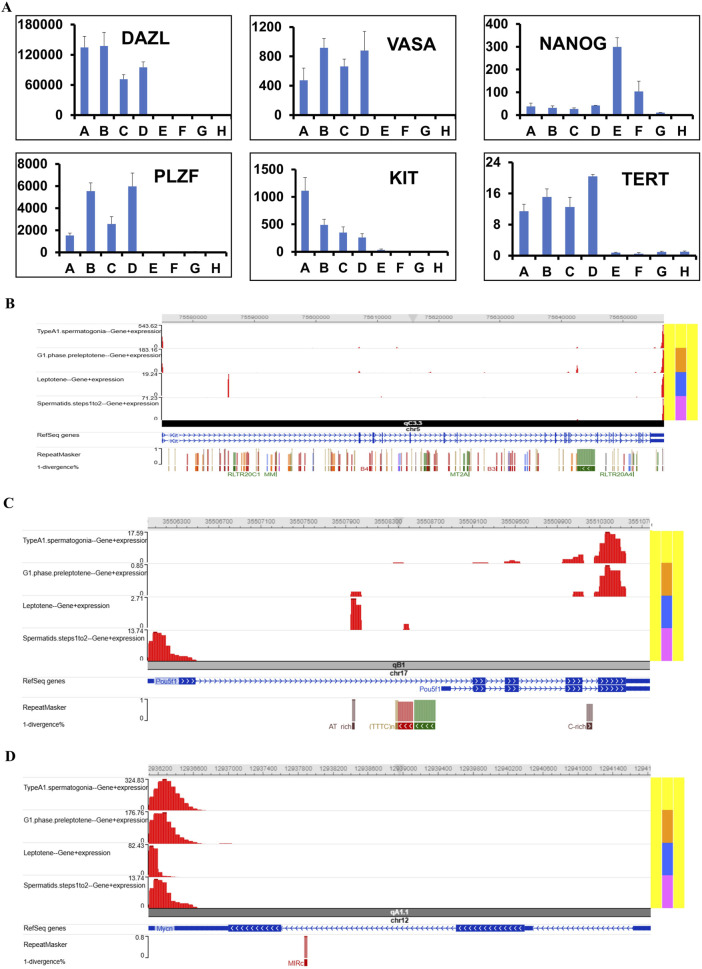
Analyzing mRNA levels quantitatively using scSeq and Fluidigm real-time RT-PCR. **(A)** The analysis revealed significantly different mRNA expression levels of Dazl, Vasa, Nanog, Plzf, Kit, and Tert in undifferentiated spermatogonia compared to differentiating, underscoring their roles in spermatogenesis (GSCs-short: A, GSCs-N: B, GSCs-2W: C, GSCs-4w: D, GPS: E, mESCs: F, TSCs: G and MEF: H). The purpose of this research is to confirm the presence of hub genes in a collection of single-cell transcriptomes derived from SSCs. Various cell types, denoted by different colors, were shown on the screen. Based on the UMAP projections, we can see that different kinds of SSC cells have the hub gene. Visualizations of the genome using scSeq in the **(B)** Kit, **(C)** Pou5f1, and **(D)** Nmyc programs for the marker genes in scRNA-seq.

In the first and ninth passages of undifferentiated SSCs, ES-like cells, mES, and epiblast cells, the mRNA expression level was quantified using Fluidigm real-time RT-PCR for the Dazl, Vasa, Nanog, Plzf, Kit, and Tert genes. The expression of NANOG increases as the number of passages increases, as shown by comparing the mRNA levels of Dazl, Vasa, Nanog, Plzf, Kit, and Tert in passages one and nine of undifferentiated SSCs (Figure 7).

### Gene co-expression

**FIGURE 8:**
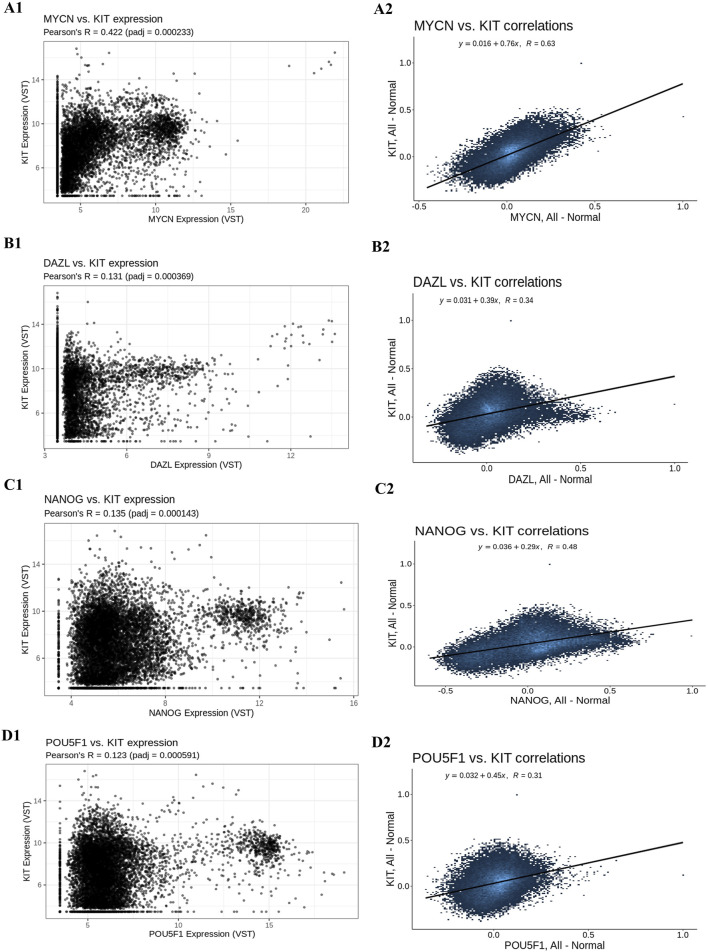
Gene co-expression analysis. (A1) Gene’s co-expression between Kit and Nmyc, (A2) correlation between Kit and Nmyc, (B1) Gene’s co-expression between Kit and Dazl, (B2) correlation between Kit and Dazl, (C1) Gene’s co-expression between Kit and Nanog, (C2) correlation between Kit and Nanog and (D1) Gene’s co-expression between Kit and Pou5f1, (D2) correlation between Kit and Pou5f1.

After removing the four outlier samples from further analysis, the sample clustering was determined to be 15 ° in height. In the modules Kit, 18 hub genes and their co-expression partners—Nmyc (Figure 8A), Dazl (Figure 8B), Nanog (Figure 8C), and Pou5f1 (Figure 8D)—were discovered using the criteria of MM > 0.9 and GS > 0.2.

### Cell–cell interactions using ligand–receptor interactions

**FIGURE 9:**
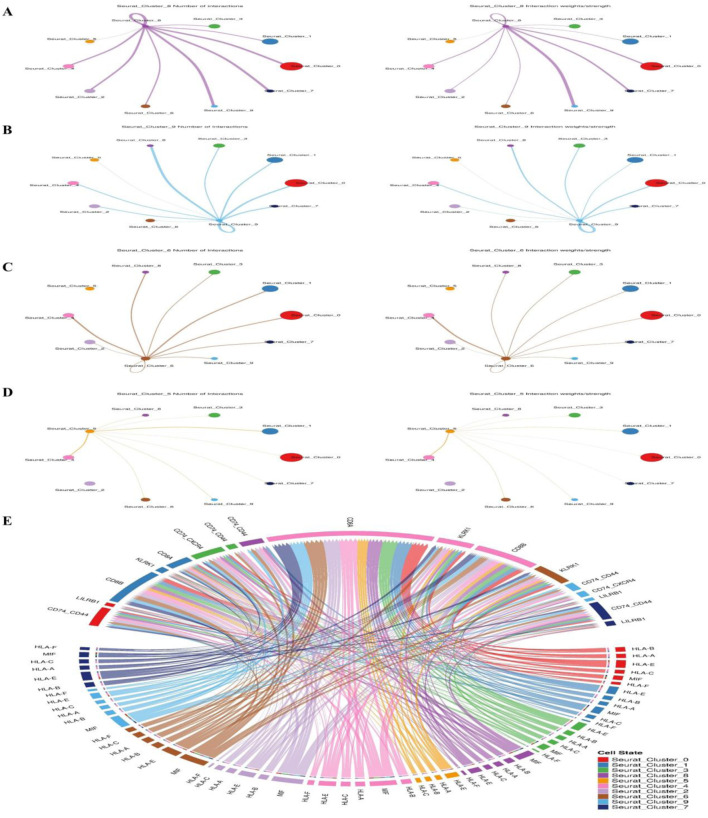
Research using single-cell RNA sequencing in both adult and pup spermatogonia from mice shows that the two species share a Kit signaling mechanism. The Kit signaling pathway is associated with **(A)** cell cluster markers, **(B)** pluripotency and the Wnt signaling pathway, **(C)** the regulation of interleukin-3, **(D)** the receptor for stem cell factors, and **(E)** cell-cell communication.

After a number of cell types were identified, we moved on to evaluate possible linkages between all of the cell types in the spermatogenesis microenvironment. We drew from a database of over 2,500 verified and supported by science interactions. Given their significance in cancer immunology, these interactions encompass receptor-ligand interactions from various families, such as the Wnt signaling pathway and pluripotency, the transcriptional targets of the Kit receptor, the regulation of interleukin-3, and signaling events mediated by the stem cell factor receptor (c-Kit). By looking for cases when different cell types within the tumor microenvironment expressed both parts of a certain ligand-receptor interaction, we were able to find similar cell-cell interactions in each of the six syngeneic tumor models (Figure 9). To score the connections, researchers took the average expression levels of both the receptor and the ligand in the cell types under study and multiplied them together. This method is called scoring ligand-receptor interactions. We used the average expression value for every cell type to reduce the possibility of false negatives caused by zero dropouts. We calculated the mean interaction score across all tumor models by assigning scores to each tumor. Because of this, we were able to identify preserved relationships. Following the analysis of numerous cell-cell interactions, we used a one-sided Wilcoxon rank-sum test and the Benjamani-Hochberg multiple hypothesis correction to determine the statistical significance of each interaction score. This analysis included 64 cell type combinations and approximately 1,200 ligand-receptor pairs that were translated to mouse homologs. We looked at interactions involving all known cell types, but we zeroed in on cases where macrophages or cancer-associated fibroblasts (CAFs) released the ligand. This choice was made because different ligands mostly originate from these two cell types. On top of that, we checked every possible contact between tumor cells (Figure 9).

## Discussion

The study on mouse spermatogonia selection and culture provides significant insights into the isolation and characterization of SSCs. The use of CD49f-MACS and matrix selection techniques effectively enriched spermatogonial populations, as evidenced by the consistent expression of markers such as Dazl, Pou5f1 (Oct4), Gfra1, and Nanog. These findings align with previous research highlighting the importance of these markers in identifying undifferentiated spermatogonia. Recent advancements have further elucidated the molecular mechanisms governing SSC maintenance and differentiation. For instance, studies have demonstrated the role of CHD4 in SSC self-renewal, revealing its interaction with key transcription factors that drive this process. Additionally, the construction of PPI networks has identified critical genes essential for SSC self-renewal, providing a comprehensive understanding of the intracellular signaling pathways involved. The functional enrichment analysis of DEGs in the current study underscores the involvement of pathways such as the Wnt signaling pathway and pluripotency, Kit receptor transcriptional targets, and interleukin-3 regulation. These pathways have been implicated in previous research as pivotal in regulating SSC functions. For example, the Wnt signaling pathway is known to play a crucial role in stem cell maintenance and differentiation.

The c-kit gene encodes a receptor tyrosine kinase essential for various stages of spermatogenesis. Recent studies have provided deeper insights into its expression patterns, regulatory mechanisms, and functional significance in germ cell development. A study by Zhang et al. examined c-kit expression during SSC differentiation. The researchers observed dynamic changes in c-kit transcription and translation before and after SSC differentiation. Notably, retinoic acid (RA) was identified as a significant upstream regulator, enhancing c-kit expression and promoting SSC differentiation (Zhang et al., 2013). This finding underscores the pivotal role of RA in modulating c-kit-mediated pathways during spermatogenesis. In a comprehensive analysis of gene expression during mammalian spermatogenesis, Green et al. reported that c-kit expression persists into the preleptotene stage of meiosis. This sustained expression suggests that c-kit not only influences spermatogonial differentiation but may also play a role in the early meiotic processes, highlighting its multifaceted involvement in germ cell maturation (Green et al., 2018).

Furthermore, research by Busada et al. (2015) demonstrated that RA regulates c-kit translation during spermatogonial differentiation. While prospermatogonia and undifferentiated spermatogonia transcribe the c-kit gene, translation of the resulting mRNAs is inhibited until differentiation is initiated by RA. This post-transcriptional regulation ensures that c-kit protein is produced at the appropriate developmental stage, preventing premature differentiation.

These studies collectively highlight the critical role of c-kit in spermatogenesis, particularly in the differentiation of spermatogonia and the initiation of meiosis. The regulation of c-kit by factors such as retinoic acid underscores the complex interplay of signaling pathways necessary for proper germ cell development (Zhao Y. et al., 2024). Understanding these mechanisms provides valuable insights into male fertility and potential therapeutic targets for infertility treatments (Liu et al., 2024). Furthermore, the application of WGCNA has facilitated the identification of functional gene modules associated with SSC characteristics. Recent studies employing single-cell RNA sequencing have provided deeper insights into the gene regulatory networks governing SSC development, highlighting the conservation of key transcription factors across species (Davoodi Nik et al., 2024; Hashemi Karoii and Azizi, 2022).

Recent hot topics in SSC research include the development of xeno-free, chemically defined culture systems, *in vitro* spermatogenesis, and the use of 3D organoids and testicular organ-on-chip platforms to more accurately replicate the SSC niche (Nguyen et al., 2024). Despite advances, key challenges remain, including the long-term maintenance of SSCs, prevention of spontaneous differentiation, and functional transplantation potential, particularly in human models (Kaltsas et al., 2025). In parallel, the rise of omics technologies—especially scRNA-seq, epigenomic profiling (e.g., ATAC-seq, DNA methylation studies), proteomics, and multi-omics integration—has provided unprecedented resolution into SSC heterogeneity and developmental trajectories (Kaltsas et al., 2025). Studies have begun to map SSC subpopulations, uncover cell fate transitions, and identify regulatory elements driving SSC maintenance and differentiation. However, integrated multi-omics data across developmental timepoints and microenvironmental contexts are still limited.

In comparison, the present study combines efficient SSC enrichment (via CD49f-MACS and matrix selection) with microarray-based transcriptome profiling, complemented by external scRNA-seq datasets and PPI network construction. This approach identifies key regulatory pathways (e.g., Wnt signaling, KIT/RA axis) and transcriptional hubs involved in SSC biology. While not a multi-omics study *perse*, it lays the groundwork for future integrative analyses (Salem et al., 2023).

The investigation of cell–cell interactions through ligand–receptor analyses has revealed intricate communication networks within the SSC niche. For instance, the interaction between pleiotrophin (PTN) from Leydig cells and its receptor syndecan-2 (SDC2) on SSCs has been identified as a regulatory mechanism in mouse spermatogonial development (Zhao X. et al., 2024). These findings emphasize the complexity of the SSC microenvironment and the necessity of considering these interactions in culture systems. *In vitro* culture systems have been instrumental in studying SSC biology. Recent efforts have focused on optimizing culture conditions to support SSC maintenance and differentiation. For example, the use of decellularized testicular matrix hydrogel scaffolds has been explored to enhance the differentiation of mouse spermatogonia, providing a more physiologically relevant environment. ​ Despite these advancements, challenges remain in replicating the complex *in vivo* environment of SSCs. The development of three-dimensional culture systems and the incorporation of key niche factors are areas of ongoing research aimed at improving SSC culture outcomes. Additionally, the translation of findings from mouse models to human applications necessitates further investigation, particularly in understanding species-specific differences in SSC biology.

## Conclusion

This study provides valuable insights into the selection, culture, and molecular characterization of spermatogonia, with a particular focus on the role of key regulatory genes such as Kit, Nanog, Dazl, and Pou5f1. The results highlight that SSCs maintain a distinct morphological and molecular profile, which is critical for their self-renewal and differentiation. PPI network and gene ontology enrichment analyses further emphasize the involvement of these genes in pathways regulating stem cell pluripotency, differentiation, and proliferation. Among these, Kit (c-kit) emerged as a pivotal factor in spermatogenesis, corroborated by recent studies demonstrating its role in SSC differentiation and early meiosis initiation. Research indicates that Kit expression is modulated by RA, ensuring its activation at the appropriate developmental stage to promote spermatogonial transition. Additionally, single-cell RNA sequencing analysis and co-expression studies reveal strong associations between Kit, Nmyc, and other stem cell markers, reinforcing its essential function in germ cell lineage commitment.

## Data Availability

The original contributions presented in the study are included in the article/Supplementary Material, further inquiries can be directed to the corresponding author.
